# Renal metastasis after esophagectomy of esophageal squamous cell carcinoma: a case report and literature review

**DOI:** 10.1186/1477-7819-12-165

**Published:** 2014-05-26

**Authors:** Yan Sun, Xinmin Yu, Yiping Zhang

**Affiliations:** 1 Department of Chemotherapy, Zhejiang Cancer Hospital, 38 Guangji Road, 310022 Hangzhou, P.R. of China; 2 Key Laboratory Diagnosis and Treatment Technology on Thoracic Oncology, Zhejiang, Province Hangzhou 310022, China

**Keywords:** Kidney, Metastasis, Esophageal carcinoma

## Abstract

Solitary metastatic renal tumors are rarely encountered. We report the case of a 63-year-old man who developed a solitary renal metastasis after undergoing an esophagectomy for esophageal squamous cell carcinoma and subsequent nephrectomy of the right kidney.

## Background

Esophageal cancer (EC) is one of the top six leading causes of death from cancer; it exhibits a strikingly uneven geographical distribution, resulting in focal endemic high-incidence areas in several countries [1]. Although considerable advances in diagnosis, surgical techniques and chemoradiotherapy have been made, EC still remains one of the most lethal cancers and most patients die from its recurrence or metastasis, with a five-year survival rate as low as 16% in 2009 in the United States [2]. In China, the situation is even worse [3]. Although 50% to 70% of patients can be treated surgically with a chance of cure, half of the patients suffer from local recurrences or metastasis after complete resection. The most common sites of metastasis are the liver, lung, bone and adrenal glands [4]. Metastases of esophageal carcinoma in the kidney are considered to be extremely rare, especially a unilateral renal metastasis [5]. There are few cases that have been reported of solitary renal metastasis of an esophageal carcinoma [6-12]. Herein, a case of solitary, unilateral renal metastasis in a patient after esophagectomy is reported (Table 1).

**Table 1:** Reported cases of metastatic renal tumor of esophageal cancer

**Case**	**Sex**	**Age**	**Side**	**Histology**	**Treatment**	**OS after renal metastasis (month)**
1 [5]	Male	64	Left	SCC	Nephrectomy	Unknown
2 [10]	Male	65	Right	SCC	Nephrectomy + Chemotherapy	6+
3 [6]	Male	57	Right	SCC	Nephrectomy	3+
4 [6]	Male	57	Right	SCC	Nephrectomy	2
5 [7]	Male	61	Left	SCC	Nephrectomy + Chemotherapy	2
6 [8]	Male	74	Right	SCC	Partial nephrectomy	Unknown
7 [9]	Male	50	Right	SCC	Nephrectomy + radiotherapy	4
8 [11]	Male	62	Left	SCC	Chemotherapy + irradiation + nephrectomy	Unknown
9 [12]	Male	56	Left	Epidermoid	Nephrectomy	6
10 [14]	Male	62	Left	Epidermoid	Nephrectomy	2

OS, overall survival; SCC, squamous cell carcinoma.

## Case report

**Figure 1:**
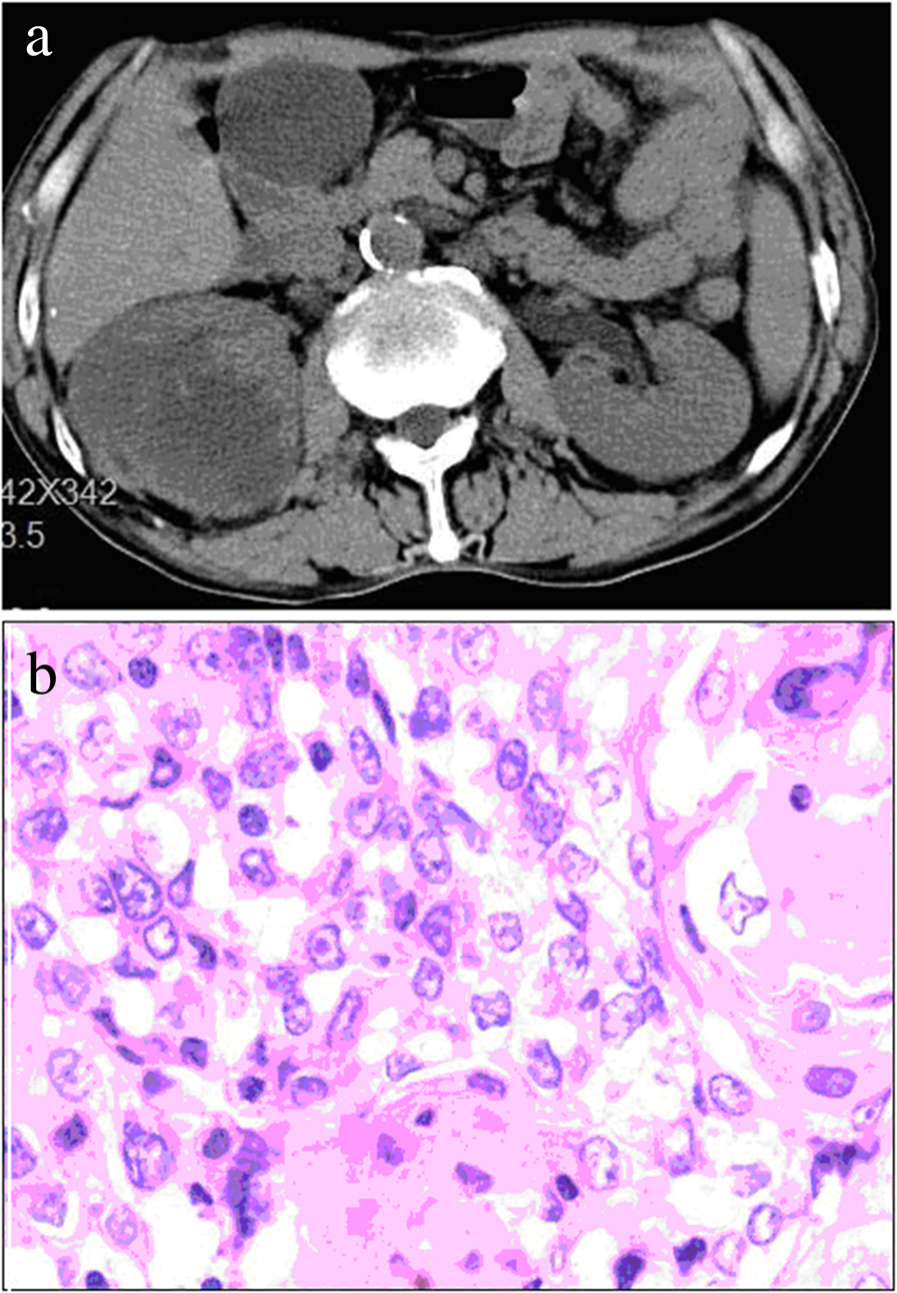
**Abdominal CT scan and histological showing a kidney metastasis of esophagus cancer. a)** Abdominal CT scan showing a mass in the right kidney. **b)**‘Histological examination revealed the same cell type as the previous esophagus cancer. (H&E, 100).

A 63-year-old man was admitted to our hospital for an evaluation of progressive dysphagia for about one month. A barium esophagogram demonstrated a mass in the low esophagus. Chest and abdominal computed tomography (CT) showed no abnormalities in other organs. The patient underwent esophagectomy with stage P-T2N0M0 in December 2008. Pathological examination of the specimen revealed squamous cell carcinoma with moderate differentiation. The patient was referred again for osphyalgia nine months after the esophagectomy, with no hematuria or any other urinary symptoms. Abdominal CT and ultrasound examination showed a mass measuring 6.5 × 1.5 cm in the right kidney (Figure 1a). No metastatic lesions were evident at other sites. A right nephrectomy was performed. Pathological examination of the specimen led to a diagnosis of squamous cell carcinoma (Figure 1b).

The patient died of whole body metastasis and respiratory failure in December 2009.

## Discussion

The incidence of distant metastases from a resectable esophageal carcinoma has been reported as approximately 20% to 30% [13]. Several reports demonstrated that the most common site of spread is the abdominal lymph nodes, followed by the liver, lung and bone. Autopsy studies have shown that about 12% of patients dying of cancer have renal metastases, making the kidney a common metastatic site [14]. In contrast to autopsy findings, clinical detection of these tumors is rare. The reason may be attributable to ignorance of such rare metastases while the patient is alive. Most patients with renal metastases are asymptomatic, despite extensive renal involvement. Hematuria and proteinuria occur in fewer than 20% of patients. Therefore, the diagnosis of renal metastases is very difficult and it is often found accidentally.

There are some differences in clinical manifestation between primary renal cell carcinoma and metastatic carcinoma. Most primary renal cell carcinomas are, on average, larger than metastatic renal tumors. Metastatic renal tumors are frequently observed in subcapsular locations. The typical pattern of renal metastases consists of multiple small nodules and almost all cases are associated with widespread non-renal metastases [15]. Moreover, unilateral, solitary renal metastasis is extremely rare. With respect to the pathology, the clear cell type is most commonly observed in primary renal cell carcinoma. However, squamous cell carcinoma has the highest occurrence among metastatic renal tumors from esophageal carcinoma.

Because of the rarity of renal metastasis of esophageal carcinoma, no conclusive treatment has been established yet according to the European Association of Urology (EAU) guidelines and National Comprehensive Cancer Network (NCCN) guidelines. Imada *et al*. [16] suggested that an aggressive and careful surgical approach with adequate follow-up may offer a chance of long-term survival for patients with multiple cancers. Grise *et al*. [12] reported that solitary metastatic renal tumor could be an indication for nephrectomy. In our case, since the tumor seemed to be a solitary renal metastasis without any other metastatic lesions, nephrectomy was performed. Pathological assessment showed a single metastatic squamous carcinoma of the same cell type as the previous esophagus cancer. The present case serves to demonstrate that careful follow up is needed for esophageal cancer patients with a cancer in another organ including rare site metastasis.

The median survival following the detection of a recurrent or metastatic esophageal carcinoma is two to ten months [17]. The treatment outcome of a recurrent disease is disappointing, and the prognosis is poor. In this case, the patient achieved a nine-month recurrence free survival time after esophagectomy, and recurred with a solitary metastasis mass in the right kidney. A nephrectomy was performed and the patient died of metastasis three months later.

## Conclusions

In summary, a renal metastasis after anesophagectomy of esophageal carcinoma is reported. Renal metastasis should be considered whenever a mass in the kidney is identified. The prognosis is poor even when surgery is performed.

## Consent

Written informed consent was obtained from the patient for the publication of this report and any accompanying images.

## Competing interests

The authors declare that they have no competing interests.

## Authors’ contributions

XY and YZ cooperated in the conception and design of the study, and in the collection of the data; YS drafted the manuscript. All authors approved the final manuscript.
